# The Effect of Material Fresh Properties and Process Parameters on Buildability and Interlayer Adhesion of 3D Printed Concrete

**DOI:** 10.3390/ma12132149

**Published:** 2019-07-04

**Authors:** Biranchi Panda, Nisar Ahamed Noor Mohamed, Suvash Chandra Paul, GVP Bhagath Singh, Ming Jen Tan, Branko Šavija

**Affiliations:** 1 Singapore Centre for 3D Printing, School of Mechanical & Aerospace Engineering, Nanyang Technological University, 50 Nanyang Avenue, Singapore 639798, Singapore; 2 Discipline of Civil Engineering, School of Engineering, Monash University Malaysia, Bandar Sunway, Selangor 47500, Malaysia; 3 Laboratory of Construction Materials, IMX, EPFL, 1015 Lausanne, Switzerland; 4 Microlab, Faculty of Civil Engineering and Geosciences, Delft University of Technology, 2628CN Delft, The Netherlands

**Keywords:** 3D concrete printing, thixotropy, bond strength, process parameters

## Abstract

The advent of digital concrete fabrication calls for advancing our understanding of the interaction of 3D printing with material rheology and print parameters, in addition to developing new measurement and control techniques. Thixotropy is the main challenge associated with printable material, which offers high yield strength and low viscosity. The higher the thixotropy, the better the shape stability and the higher buildability. However, exceeding a minimum value of thixotropy can cause high extrusion pressure and poor interface bond strength if the printing parameters are not optimized to the part design. This paper aims to investigate the effects of both material and process parameters on the buildability and inter-layer adhesion properties of 3D printed cementitious materials, produced with different thixotropy and print head standoff distances. Nano particles are used to increase the thixotropy and, in this context, a lower standoff distance is found to be useful for improving the bond strength. The low viscosity “control” sample is unaffected by the variation in standoff distances, which is attributed to its flowability and low yield stress characteristics that lead to strong interfacial bonding. This is supported by our microscopic observations.

## 1. Introduction

In the last few decades, a lot of attention has been paid to sustainability in the built environment. In that context, 3D concrete printing (3DCP) is a rapidly developing field. This is illustrated by the growing number of projects in both private enterprises and research institutes worldwide. The expected benefits of 3DCP are higher productivity, faster construction processes, higher geometrical freedom, and lower production costs [1,2,3]. In spite of a growing number of researchers active in this field, fundamental scientific understanding of the relationship between design, material, and process parameters is limited. The literature reveals that both printability and post-print properties (e.g., interface bond strength) are highly dependent on the material rheology and the process parameters, such as printing speed, time, temperature, etc. [4,5]. Consequently, significant research efforts have been devoted to tuning the material’s early-age properties [6,7] and printing parameters [8]. A soft, flowable material is easy to extrude. On the other hand, a stiff material can sustain the weight of more layers, although it needs more pressure for the extrusion. However, the stiffness of a cementitious material changes with time due to ongoing hydration. As a result, it may not be extrudable after its dormant period. In addition, this sometimes results in poor interface bond strength due to lack of moisture, mechanical contacts, and the presence of pores in the substrate [9,10,11,12,13,14].

Panda et al. [15] studied the effects of the time gap and standoff distance on the inter-layer bond strength of 3D printed geopolymer concrete. The experimental results agreed well with the findings of Le et al. [16] who observed a decrease in bond strength with increasing the time gap due to a reduction in layer adhesion. In an extensive experimental study, Wolfs et al. [8] reported similar findings. According to Sanjayan et al. [17], loss of surface moisture is one of the major factors affecting the inter-layer strength, in addition to process parameters, evaporation rate, and bleeding rate. Roussel [18] confirmed the impact of moisture loss while comparing the bond strength of an interface protected from drying with one exposed to drying. He found that the interface strength was 90% higher when the material was protected from drying compared to the reference material. Tay et al. [19] studied the effect of time-dependent rheological behavior of printable materials. They attributed the low bond strength to the increase in storage modulus (G’) of the first layer with increasing the time gap. Nerella et al. [20] attributed the poor bond strength to micro-pores formed in the interlayers of 3D printed samples by prolonged time gaps between consecutive layers.

Recently, efforts have been made to improve the bond between consecutive layers by increasing the mechanical contact between the layers [21] or spraying fresh cement paste in the interfacial zone prior to extrusion of the subsequent layer [22]. Nevertheless, it is still necessary to investigate the origins of bond strength between the layers in 3D printed concrete. Furthermore, quantification of the bond strength according to the material design required for concrete printing is needed. Therefore, this paper studies the effects of material’s fresh properties (yield stress, thixotropy) and print head standoff distance (SD) on 3D printability and the bond strength of fly ash based cementitious mixtures modified with nanoclay. Nanoclay is added to improve the thixotropy of the material, and its effect on both buildability and bond strength is evaluated with different time gap intervals. In addition, the microstructural features of the interface are observed using optical microscopy. Reaction products in the material are examined using X-ray diffraction (XRD) and scanning electron microscopy (SEM).

## 2. 3D Concrete Printing and Related Process Parameters

**Figure 1:**
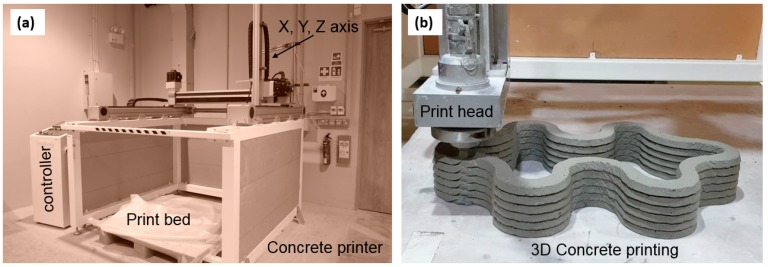
3D concrete printing (3DCP) equipment at Nanyang Technological University (NTU), Singapore: (**a**) 4-axis concrete gantry printer (**b**) 3DCP process.

The development of 3D concrete printing (3DCP) started in the mid of 1990s in California, USA, when Khoshnevis introduced the Counter Crafting technique [23], by sequentially depositing fresh concrete layers according to a digital CAD model. The main challenge in printing concrete lies in developing a thixotropic material that is easily extrudable and can withstand the loads of subsequent layers without significant deformation [24,25]. These requirements are, however, in contradiction with conventional concrete technology: “regular” concrete is designed to be pumpable or self-compacting, so that it can fill the mold without the need of additional vibration. In 3D printing, however, the material needs to retain its shape and harden quickly to support the layers deposited on top of it soon after being pumped. Due to this contradicting demand, the conventional concrete needed to be redesigned for 3D printing applications with the help of advanced additives such as superplasticizers, accelerators, retarders, or viscosity modifiers [26]. A schematic diagram of the 3DCP process, developed at Nanyang Technological University (NTU), Singapore, is shown in Figure 1.

## 3. Materials and Methods

The raw materials used in this study were Class F fly ash (FA) conforming to ASTM C 618 [27] and CEM 1 Portland cement (OPC) (LafargeHolcim, Singapore). Micro silica (SF) (Elkem Pvt. Ltd., Singapore) was added by partially replacing the FA, while the water/binder ratio was kept constant at 0.35. According to the authors’ previous study [28], 30% OPC was used with 67.5% FA and 2.5% SF (by mass) as the control mix (CM) that satisfies the required properties of 3D printable materials. Additionally, 0.5 wt % (of binder) nanoclay (Actigel^®^, Active Minerals International, Eatonton, GA, USA) was incorporated in the CM mix to enhance the green strength (yield strength) by homogeneously dispersing it in the required water. The clay percentage was selected based on optimum rheology properties (yield stress and viscosity) and referred as “NM” mix. As suggested by Dakhane et al. [29], we used 3 wt % (of binder) reagent grade sodium sulfate (Sigma-Aldrich) activator along with fine aggregate (river sand of maximum particle size 2 mm) to formulate 3D printable high-volume fly ash mortar (sand:binder = 55:45).

### 3.1. Tensile Bond Strength Measurement

**Figure 2:**
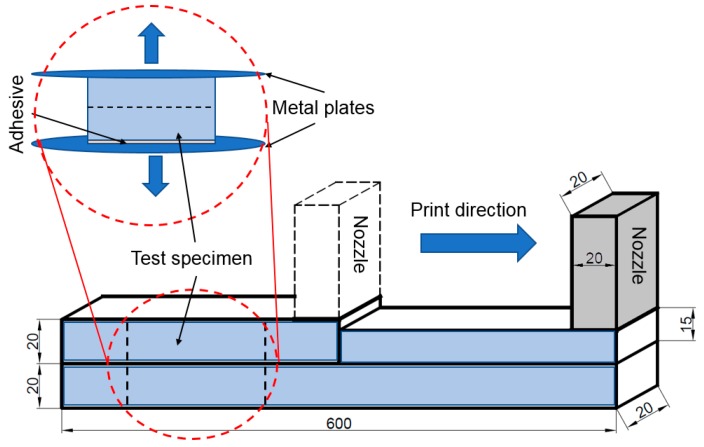
Schematic of sample preparation for tensile bond strength (dimensions are in mm).

Two layers with 600 mm length (time gap: 15 min) were printed using a 4-axis gantry printer with a square nozzle of 20 × 20 mm opening. The first 300 mm was printed with 20 mm SD, while for the remaining length a reduced SD of 15 mm was maintained as shown in Figure 2. Pump flow rate and print speed were kept at their optimum levels to ensure sound deposition of the extruded material.

The standoff distance (SD), i.e., the height of the nozzle above the print surface, has a considerable influence on the interface properties of the printed product [15]. To investigate this effect, 40 mm length specimens were extracted from the extruded filament for a tensile bond strength test. The test samples (after 28 days of ambient curing) were subjected to tensile loading at a rate of 0.035 ± 0.015 MPa/s and their average strength was calculated based on the maximum failure load and effective bonding area, as described in previous studies by the authors [15]. The fracture surfaces and inter-layer microstructure were observed in an optical microscope and their micro graphs were used for further analysis.

### 3.2. Flow Properties

A rotational rheometer (MCR 101 Anton Paar^®^, Singapore) was used to measure the yield strength of both CM and NM mixtures at different resting times (5 and 15 min). A stress growth test was adopted (0.1 s^−1^ shear rate for 60 s) and the maximum stress was counted as static yield stress as described in [30]. The thixotropy of the mixtures was quantified through a structural parameter (*λ*) [31]:

(1) $$\lambda =\;\frac{\lambda_{o}-\lambda_{e}}{\lambda_{e}}$$

where $\lambda_{e}$ and $\lambda_{o}$ are the equilibrium stress and initial shear stress, respectively. The λ value indicates the thixotropy of the mix, and was found to be higher for the NM mix [27].

### 3.3. Mechanical Properties and Microstructure Characterization

**Figure 3:**
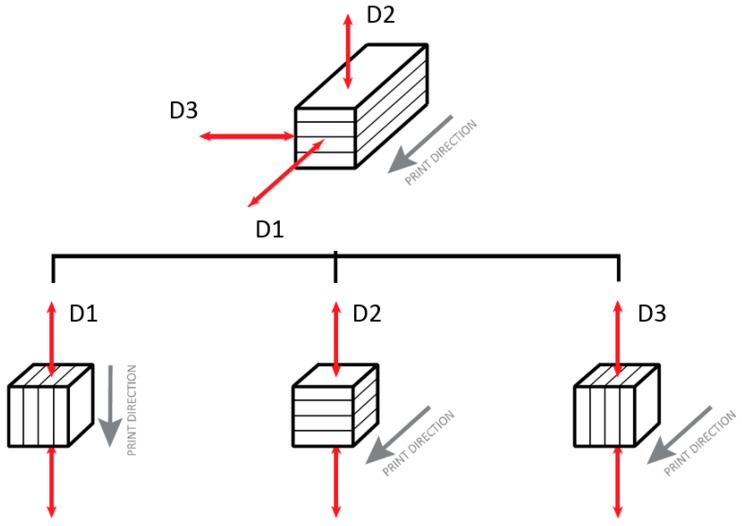
Schematic overview of testing directions for compression test.

The compressive strength of 3D printed specimens was measured using specimens of 50 × 50 × 50 mm according to BS EN 196-1:2016. An ALPHA 3-2000A machine, operated at a loading rate of 100 N/min, was used. Before the test, all samples were cured at room temperature (25 ± 2 °C) for 28 days. The influence of anisotropy caused by the printing process was assessed by testing the material in three perpendicular directions, as shown Figure 3. Fifty-millimeter cube samples were extracted from a 350 × 300 × 150 mm slab, and 3D printed using a 30 × 15 mm rectangular nozzle.

X-ray diffraction (XRD) and field emission scanning electron microscopy (Fe-SEM) were used to characterize the NM mix and the reaction products [32]. Randomly oriented powder samples were extracted from the selected mixes by grinding the dried samples for XRD analysis. The scan was measured between 10° and 70° at 0.02° step size at 0.53 steps per second. The X-ray tube generator was operated at 40 kV and 36 mA. The total time for each scan was about 30 min per scan. The external standard method was used for the quantification of the different crystalline phases [33,34]. Corundum was used as the external standard. Rietveld quantitative phase analysis was performed using TOPAS 5 software. Crystal structures of known phases were taken from the Inorganic Crystal Structure Data (ICSD) base. Pseudo Voigt was used to fit the crystalline and amorphous phases, while Chebyshev polynomial combined with a 1/2θ term was used to fit the background intensity. The quality of fit was judged by viewing the observed and calculated patterns and using the difference curve [34,35]. The bound water content, determined by the mass loss at 50–600 °C, was included in the calculation of the hydrated phases and the mass absorption coefficient of the hydrated samples. The glassy content and hydration product content were determined using the XRD based direct decomposition method [34].

For FESEM analysis, a JEOL JSM-7600F electron microscope was used to examine the sample microstructure after 28 days of ambient curing.

## 4. Results and Discussion

### 4.1. Effect of Nanoclay on Buildability and Bond Strength

**Figure 4:**
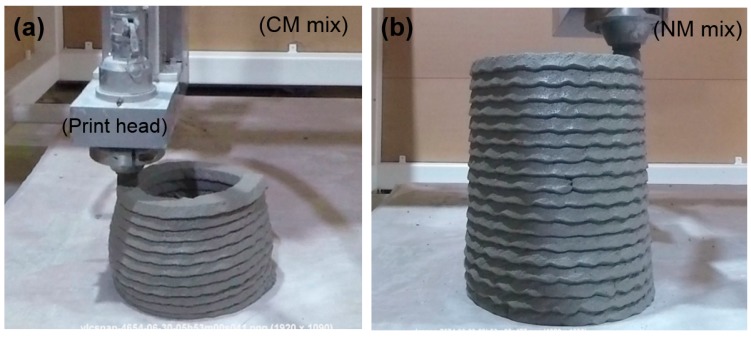
Demonstration of 3D printability of (**a**) control mix (CM); (**b**) 0.5% clay modified NM mixture.

**Figure 5:**
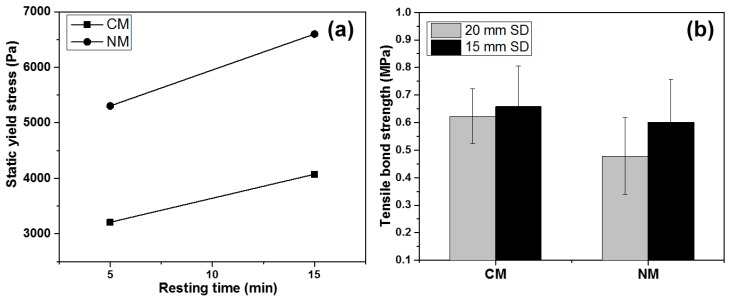
(**a**) static yield stress; (**b**) tensile bond strengths of CM and NM mixtures.

Figure 4 shows the effect of yield stress on the 3D printability of high-volume fly ash material. It was found that the NM mix has significantly higher buildability than the CM mix. This can be attributed to the high static yield stress and thixotropy of the NM mix. The yield strength of the NM mix was approximately two times higher than that of the CM mix (Figure 5a). Yield strength is one of the fresh properties of printable materials responsible for shape stability and buildability. In 3DCP, after the extrusion, there is a need for high yield strength for supporting the subsequent layers. In this work, therefore, a small amount of nanoclay was added to the CM mix to improve the yield strength and thixotropy. Nanoclay carries a negative charge on its faces and a positive charge on its ends. During the material flow, it tends to separate from each other by the electrical repulsion between similar charges. On the other hand, at rest, it flocculates by oppositely charged ends, while increasing the yield stress and viscosity [36]. It is also interesting to note that the yield strength evolution rate in both mixtures followed the same trend (as indicated by parallel lines in Figure 5a). Nevertheless, the addition of nanoclay resulted in higher initial yield strength. This is in accordance with the literature [30], where nanoclay shows an immediate effect on thixotropy and a relatively moderate growth over time. Due to the low yield strength of the CM mix, the bottom layer started to collapse after the 10th layer and expanded in a lateral direction as the vertical deformation increased. Such “bulging” type of failure is known as strength-based failure. The addition of nanoclay was able to overcome this issue due to the increase in the yield strength, as described. However, nanoclay is not useful for increasing the stiffening rate, which is the limiting factor for the buildability of large-scale 3D printing projects. Optimizing the print path or alternatively, the addition of an accelerator at the nozzle can be potentially helpful in this regard [37].

The tensile bond strength results are only shown in Figure 5b for the 15 min time gap, since the strength difference was not significant for the 5 min gap. The effect of two SDs (i.e., 20 and 15 mm) on the bond strength of the CM mix was negligible. On the other hand, the 15 mm SD notably increased the bond strength of the NM mix compared to the 20 mm SD.

**Figure 6:**
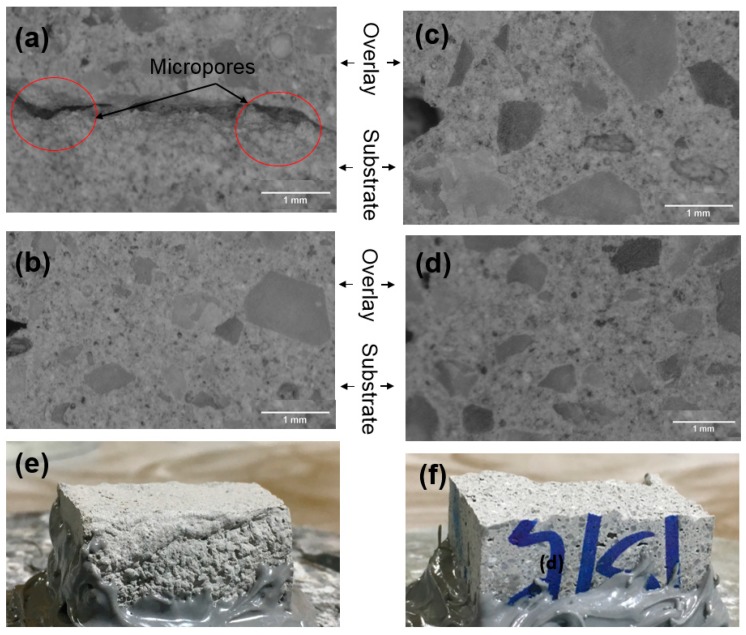
Optical microscopic images of NM mix interface for (**a**) 20 mm (**b**) 15 mm SD; Interface of CM mixture for (**c**) 20 mm and (**d**) 15 mm SD; fracture surfaces of (**e**) NM (**f**) CM mixtures for 20 mm SD.

A close look at the interface reveals the presence of macro pores (Figure 6a): this resulted in the loss of bond strength. It is known that porosity can have a marked effect on the interfacial bond [38,39]. The bond strength of the concrete-to-concrete interface is also influenced by adhesion including substrate condition, overlay compaction, and curing procedures [40,41,42,43]. More generally, an increase in porosity results in a proportional decrease of strength in quasi-brittle materials [44]. Overlay compaction, which was achieved by decreasing the SD from 20 to 15 mm, was found to improve the bond strength of the NM mix by 33%. This can be explained by a reduction in macro-porosity (Figure 6b). It is also believed that the stresses generated by the flow of the overlay layer was sufficient to initiate flow into the substrate layer, which allowed the two layers to intermix to a certain extent, as no sharp interface was visible between the layers. On the other hand, no significant impact of SDs in the case of the CM mix was observed. This can be attributed to its low yield stress. Moreover, there was no visible difference in the micrographs (Figure 6c,d) of the CM mix interface, when printed with lower SD. For 20 mm SD, the CM mix failed in the shear due to strong adhesion whereas the NM mix showed brittle failure as depicted in Figure 6e,f respectively.

The physical origin of the bond strength is also related to the stiffness (yield strength) of the substrate, which increases with the time gap. The increased yield stress and thixotropy could make the interface too stiff (less moisture) and therefore prevent intermixing with the subsequent layer. Since the overlay layers are always found to possess low yield stress (by shear force in pumping), the decrease in tensile strength could only be related to the yield stress of the substrate layer [19].

**Figure 7:**
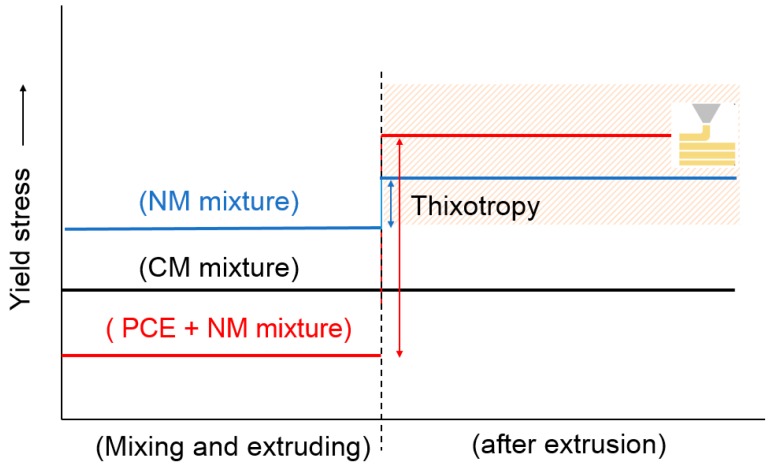
Schematic of rheology modification for 3D concrete printing.

The above results therefore confirm that high yield stress materials can improve buildability, but may result in weak interfaces while being printed with a SD equal to the nozzle width. A decrease in SD can significantly improve the interface strength or alternatively, low yield stress, thixotropic material can be used for concrete printing. However, sometimes to fulfill the buildability demand, there is a need to extrude high yield stress material, which can strongly affect the bond strength, and in this regard, a combined use of nanoclay and PCE (polycarboxylate) based superplasticizer is suggested, since PCE can effectively reduce the pumping pressure during extrusion and nanoclay can improve the yield stress after deposition without causing any process instability. Nanoclay can flocculate well in the presence of PCE admixture [45] and using such a combination, large scale concrete printing can be accomplished without compromising the buildability and inter-layer bond strength. A schematic diagram of such a combined use of admixtures and the effect is shown in Figure 7.

### 4.2. Direction of Mechanical Properties

**Figure 8:**
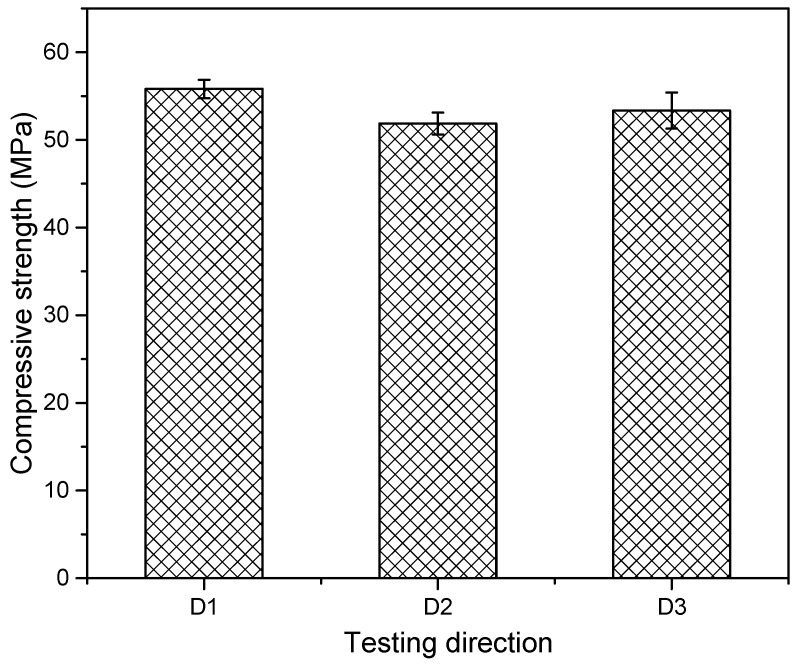
Orthotropic properties of the 3D printed NM mixture (standard deviation indicated).

Figure 8 shows a 28-day compressive strength of the NM mix for the three loading directions. It can be seen that the material is slightly stronger in D1 direction compared to the two other tested directions. The compressive strength in D2 and D3 directions were comparable despite the different printing directions. The difference in strength is certainly not associated with printing directions, since D1 and D3 have the same orientation, as shown in Figure 3. It might be possible that in D1 direction, the particles were placed and compacted better than the other directions, as the material was being deposited/extruded in the direction of D1. This anisotropy (also in terms of flexural and tensile strength) is an inherent consequence of the 3D printing process [34].

### 4.3. X-ray Powder Diffraction (XRD) and Scanning Electron Microscopy (SEM)

**Figure 9:**
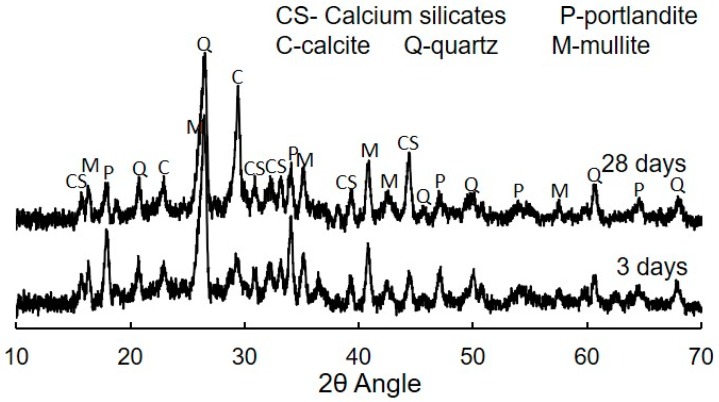
XRD spectra of the NM mix at 3 days and 28 days.

XRD is a useful tool to identify the different crystalline phases present in the material [32]. Figure 9 shows the XRD spectra of the printed NM mix at three and 28 days of hydration. For both time scales, different crystalline phases are observed along with a broad amorphous halo over the 2θ angle range. The crystalline phases are mainly associated with the calcium silicates (C_3_S and C_2_S), portlandite (ICSD #64950), calcite (ICSD #20179), quartz (ICSD #16331), mullite (ICSD #23867), along with different minor crystalline phases (not labelled in the figure due to their very low intensity). A broad halo in the diffractogram was observed between 2θ angles equal to 15° and 38°. The broad amorphous halo is the combination of the glassy phase of fly ash and the amorphous hydration products in the fly ash-cement blended system. Between 3 and 28 days, the depression in the amorphous halo at lower 2θ angles and a rightward shift of the entire halo in the diffraction pattern of the blended system, indicates a decrease in the glassy content of fly ash (centered on lower 2θ angles) and the formation of amorphous hydration products (centered on higher 2θ angles) [33,34].

**Figure 10:**
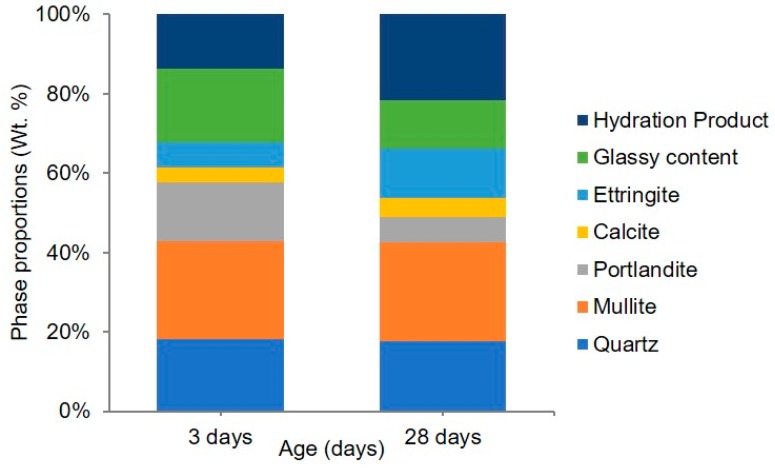
The different quantified phases of the NM mix at 3 and 28 days.

The results of the quantitative analysis of the XRD patterns via Rietveld refinement are shown in Figure 10 for 3 and 28 days. Based on the relative proportions, it can be seen that the amount of portlandite and glassy content decreases during the reaction while the amount of hydration products increases due to the pozzolanic effect of fly ash, especially in the later stage of reaction. The reduction of these is an indication of the pozzolanic reaction and is related to the material strength development, depending on the amount of hydration product formed via the synergistic effect of binary cementitious materials [34]. The mullite and quartz content did not change with age as these phases are contributed to by the fly ash and is inert in nature. The ettringite content was changing during the hydration reaction, which originated from the consumption of gypsum.

**Figure 11:**
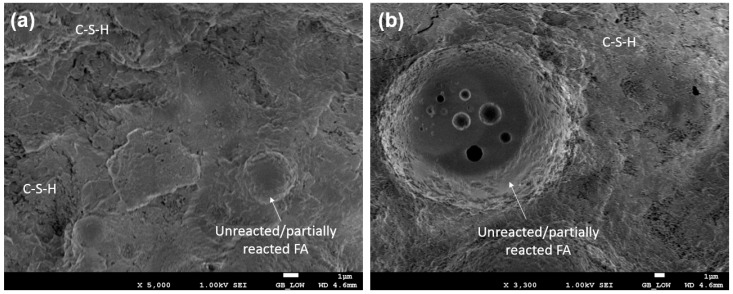
Fe-SEM image of NM mortar at 28 days showing (**a**) unreacted FA particles; (**b**) formation of C-S-H gel.

Figure 11 shows SEM images of the hardened NM mortar after 28 days of hydration at different locations. From the smooth surface of the FA, it can be deduced that some of the FA particles did not react during the hydration process and only served as an inert filler. However, the presence of sodium sulfate promoted the pozzolanic reaction of FA and, therefore, dense amorphous gel was observed with a much lower trace of CH crystals. It is considered that the majority of CH content had reacted with the amorphous silica of the FA to produce secondary calcium silicate hydrate (C-S-H) gel [46]. The denser microstructure is most likely associated with the C-S-H gel and/or filler effect of the nano particles.

## 5. Conclusions

In this article, 3D printability and the inter-layer bond strength of cementitious material were investigated with respect to the material’s early age properties and process parameters, accompanied by microscopic analysis. Based on the presented results, the following conclusions can be drawn:

- High volume fly ash material, including a very small amount of nanoclay (0.5%) shows significantly higher buildability compared to the control mix, activated with alkali sulfate salt. The improved performance is associated with the thixotropic property of clay particles, responsible for better early age mechanical properties such as yield stress and stiffness.
- The presence of macro pores is the main reason for the weak interfacial bond strength of the 3D-printed samples. This is more pronounced for the material with higher thixotropy (NM mix) and for a longer time gap between the layers.
- Reducing the standoff distance below the nozzle opening size (width) had a positive impact on improving the bond strength of the NM mix, which can be explained by the decreased porosity in the interface zone. The impact of the nozzle standoff distance was found to be more pronounced for the material with a higher yield stress value.
- The control mix, due to its low yield strength, showed comparable bond strength despite the change in standoff distances. It is considered that low yield strength material can be easily mixed with its previous layer which minimizes the pore formation, as observed in optical micrographs.
- The nanoclay modified high volume fly ash mix (NM) showed anisotropic mechanical properties, unlike conventional cast concrete. The highest compressive strength was measured when the 3D printed mortar was tested in the direction of layer deposition.
- The hydration products of the NM mix mainly consist of ettringite, portlandite, and C-(A)-S-H gel, similar to the conventional cement-based materials. Therefore, the long-term performances are stable and reliable.

In future work, other parameters such as structuration rate, roughness, environmental conditions, and the effect of chemical additives will be studied to gain new insights into interlayer bond strength in the concrete printing process.
